# Draft genome sequence of *Gracilimonas* sp. strain BCB1 isolated from the gill tissue of the lucinid bivalve *Stewartia floridana* in Pinellas County, Florida, USA

**DOI:** 10.1128/mra.00595-25

**Published:** 2025-09-26

**Authors:** Shen Jean Lim, Ojas Natarajan, Jewelia Keller, Larry J. Dishaw, Bradley T. Furman, Mya Breitbart

**Affiliations:** 1 College of Marine Science, University of South Florida 214549, St. Petersburg, Florida, USA; 2 Department of Pediatrics, Morsani College of Medicine, University of South Florida 33697, Tampa, Florida, USA; 3 Fish and Wildlife Research Institute, Florida Fish and Wildlife Conservation Commission, Saint Petersburg, Florida, USA; Portland State University, Portland, Oregon, USA

**Keywords:** seagrass, lucinid, bivalve, Florida, *Gracilimonas*, bacteria, gill, prokaryote

## Abstract

We describe the genome of *Gracilimonas* sp. strain BCB1 isolated from the gill tissue of the lucinid bivalve *Stewartia floridana* inhabiting a seagrass bed in Boca Ciega Bay, Pinellas County, Florida, USA. Genetically similar bacteria were also isolated from all other lucinid specimens collected from Pinellas County.

## ANNOUNCEMENT

Lucinidae bivalves provide ecological benefits in seagrass habitats through a tripartite symbiosis with their chemosynthetic gill endosymbionts and surrounding seagrasses (1). Nevertheless, bacterial members within lucinid gills have not yet been cultured. Live lucinids were collected by hand digging and stored in Whirl-Pak Bags (Filtration Group) containing surface water from each site (Table 1). From each specimen, one gill was removed and rinsed with 1 mL of 0.2 μm-filtered 35 g/L Instant Ocean solution (https://www.instantocean.com), homogenized with a plastic pestle in 1 mL of filtered Instant Ocean, then diluted 10× (v/v) in filtered Instant Ocean. 200 µL of the homogenate was spread on BD Difco Marine Agar 2216 with sterile glass plating beads and incubated in the dark at room temperature (~22°C).

**TABLE 1:** Metadata for each lucinid specimen collected for culturing^*a*^

Specimen ID	Collection date	Location	Latitude	Longitude	Species
SL1	9/7/23	Terra Ceia Aquatic preserve	27.587464	−82.623236	*Lucinisca nassula*
SS1	9/7/23	Terra Ceia Aquatic preserve	27.587404	−82.623248	*Stewartia floridana*
**LUC36**	**10/2/23**	**Boca Ciega Bay (BCB11**)	**27.72252**	**−82.69782**	***Stewartia floridana***
LUC37	10/2/23	Boca Ciega Bay (BCB11)	27.72252	−82.69782	*Stewartia floridana*
LUC38	10/2/23	Boca Ciega Bay (BCB11)	27.72252	−82.69782	*Stewartia floridana*
LUC39	10/2/23	Boca Ciega Bay (BCB11)	27.72252	−82.69782	*Stewartia floridana*
LUC7	10/2/23	Boca Ciega Bay (BCB17)	27.72693	−82.7293	*Stewartia floridana*
LUC8	10/2/23	Boca Ciega Bay (BCB17)	27.72693	−82.7293	*Stewartia floridana*
LUC9	10/2/23	Boca Ciega Bay (BCB17)	27.72693	−82.7293	*Stewartia floridana*
LUC10	10/2/23	Boca Ciega Bay (BCB17)	27.72693	−82.7293	*Stewartia floridana*
LUC11	10/2/23	Boca Ciega Bay (BCB17)	27.72693	−82.7293	*Stewartia floridana*
LUC12	10/2/23	Boca Ciega Bay (BCB17)	27.72693	−82.7293	*Stewartia floridana*
LUC13	10/2/23	Boca Ciega Bay (BCB17)	27.72693	−82.7293	*Stewartia floridana*
LUC14	10/2/23	Boca Ciega Bay (BCB17)	27.72693	−82.7293	*Stewartia floridana*
LUC15	10/2/23	Boca Ciega Bay (BCB9)	27.736759	−82.702695	*Stewartia floridana*

^*a*^ The specimen from which the draft genome was sequenced is highlighted in bold.

Pink colonies, comprising rod-shaped, gram-negative, oxidase-positive, and endospore-stain-negative bacteria, grew on all plates after 2 days. Colony polymerase chain reaction (PCR) was performed using universal primers 27F (5′-AGAGTTTGATCATGGCTCA-3′) and 1492R (5′-TACGGTTACCTTGTTACGACTT-3′) (2) and thermocycling conditions described in (3). PCR products were purified using Zymo Research’s DNA Clean & Concentrator-5 kit, quantified using the Qubit DNA high sensitivity (HS) assay (Invitrogen), and sequenced by Eurofins Genomics. Amplified 16S rRNA gene sequences were aligned in Unipro UGENE v.49.0 (4) and computed to be 99±2% identical to each other. BLAST searches (5) against NCBI’s nucleotide database (6) matched these sequences to *Gracilimonas sediminicola* strain CAU 1638 (GenBank accession MW132416.1) with 99% identity.

For genome sequencing, a random colony isolated from *S. floridana* was grown in BD Difco Marine Broth 2216 under the same conditions. Upon visible growth, the culture was centrifuged at 1,700 RCF for 5 minutes. DNA was extracted from the pellet using Qiagen’s AllPrep DNA/RNA Mini kit, purified using Zymo Research’s Genomic DNA Clean & Concentrator-10 kit, quantified using the Qubit DNA HS assay and the Nanodrop ND-1000 spectrophotometer, then visualized on a 1% (w/v) agarose gel. Unsheared DNA was used for library preparation by Eurofins Genomics using Oxford Nanopore Technologies’ (ONT) Rapid Barcoding Kit 96 V.14 and sequenced on a GridION R10.4.1 flow cell. Basecalling was performed using ONT’s Dorado v.0.6.0 with the dna_r10.4.1_e8.2_400bps_hac@v.4.2.0 model. Sequencing generated 139,042 raw reads with an *N_50_* value of 3,911 bp. The genome was assembled using Flye v.2.9.4 (7) implemented in KBase (8), polished using ONT’s medaka v.1.9.1, and annotated using the NCBI Prokaryotic Genome Annotation Pipeline (9). Default parameters were used unless otherwise noted.

**Fig 1:**
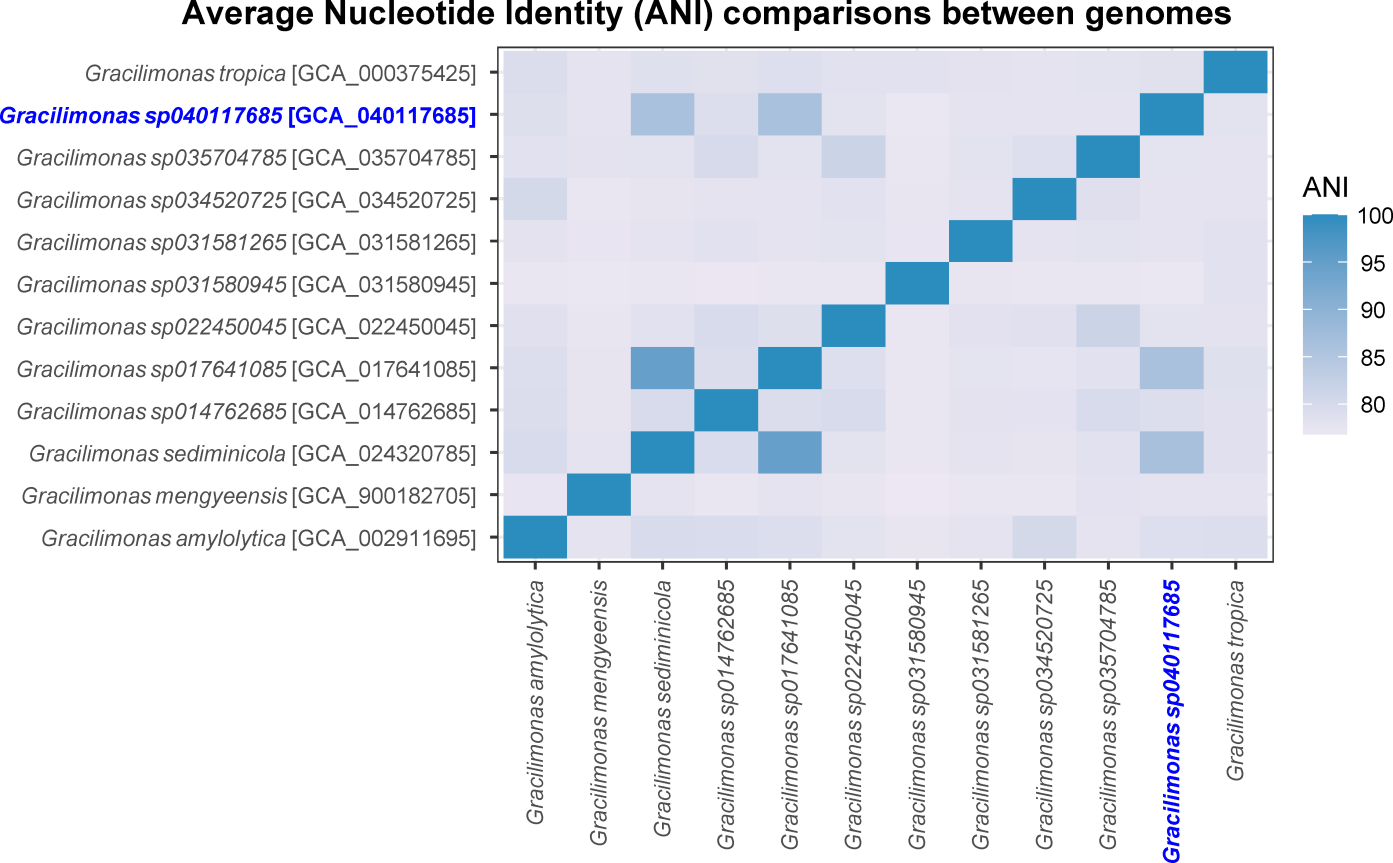
ANI comparisons between the genome of *Gracilimonas sp*. BCB1 isolated in this study assigned to *Gracilimonas sp040117685* (blue text), and other representative genomes assigned to various species under the genus *Gracilimonas* by GTDB-Tk. GenBank accession numbers are listed in square brackets.

The draft genome was 3.3 Mb with a *N_50_* value of 248.8 kb, 25 × genome coverage, and 44% G+C content. It comprises 18 contigs containing 2,957 genes and 2,886 protein-coding genes. CheckM2 v1.1.0 (10) predicted the genome to be 97.47% complete with 0.15% contamination. Using the Genome Taxonomy Database (GTDB) release R226 (11) as reference, GTDB-Tk v2.4.0 (12) assigned the genome to the species *Gracilimonas* sp040117685. The genome shared 86% average nucleotide identity (ANI) to the genome of *G. sediminicola* strain CAU 1638 (GCF_024320785.1), as computed by fastANI v.1.3 (13) (Fig. 1). Future sequencing will improve the genome assembly, while continued investigation will elucidate the physiology and ecology of this species.

## Data Availability

This Whole Genome Shotgun project has been deposited in NCBI under the BioProject accession number PRJNA1112454, RefSeq assembly accession number GCF_040117685.2, and GenBank accession number JBEDYP000000000.2. Raw reads were deposited in NCBI’s Sequence Read Archive under the accession number SRR33680651 (PRJNA1112454).
